# Myrtenol improves brain damage and promotes angiogenesis in rats with cerebral infarction by activating the ERK1/2 signalling pathway

**DOI:** 10.1080/13880209.2021.1917626

**Published:** 2021-05-19

**Authors:** Shengming Huang, Zhanguo Tan, Jirui Cai, Zhiping Wang, Yuejun Tian

**Affiliations:** a Department of Neurology, Luohe Central Hospital, Luohe City, China; b Department of Neurosurgery, Luohe Central Hospital, Luohe City, China; c Department of Cardiology, Luohe Central Hospital, Luohe City, China; d Institute of Urology, Second Hospital of Lanzhou University, Lanzhou, China

**Keywords:** Cerebral ischaemia/reperfusion, middle cerebral artery occlusion, neurological deficits, VEGF

## Abstract

**Context:**

Cerebral ischaemia/reperfusion (I/R) injury has a high disability and fatality worldwide. Myrtenol has protective effects on myocardial I/R injury through antioxidant and anti-apoptotic effects.

**Objective:**

This study investigated the effect of myrtenol on cerebral ischaemia/reperfusion (I/R) injury and the underlying mechanism.

**Materials and methods:**

Cerebral I/R injury was induced in adult Sprague-Dawley rats by middle cerebral artery occlusion (MCAO) for 90 min. MCAO rats were treated with or without myrtenol (10, 30, or 50 mg/kg/day) or/and U0126 (10 μL) intraperitoneally for 7 days.

**Results:**

In the present study, myrtenol had no toxicity at concentrations up to 1.3 g/kg. Myrtenol treatment improved neurological function of MCAO rats, with significantly (*p* < 0.05) improved neurological deficits (4.31 ± 1.29 vs. 0.00) and reduced brain edoema (78.95 ± 2.27% vs. 85.48 ± 1.24%). Myrtenol extenuated brain tissue injury and neuronal apoptosis, with increased Bcl-2 expression (0.48-fold) and decreased Bax expression (2.02-fold) and caspase-3 activity (1.36-fold). Myrtenol promoted angiogenesis in the brain tissues of MCAO rats, which was reflected by increased VEGF (0.86-fold) and FGF2 (0.51-fold). Myrtenol promoted the phosphorylation of MEK1/2 (0.80-fold) and ERK1/2 (0.97-fold) in MCAO rats. U0126, the inhibitor of ERK1/2 pathway, reversed the protective effects of myrtenol on brain tissue damage and angiogenesis in MCAO rats.

**Discussion and conclusions:**

Myrtenol reduced brain damage and angiogenesis through activating the ERK1/2 signalling pathway, which may provide a novel alternative strategy for preventing cerebral I/R injury. Further *in vitro* work detailing its mechanism-of-action for improving ischaemic cerebral infarction is needed.

## Introduction

Cerebral infarction (CI), also known as cerebral ischaemia stroke, is mainly caused by focal cerebral ischaemia/reperfusion (I/R) injury, with high disability and lethality worldwide (Iizuka et al. 2019). Numerous studies have demonstrated that multiple physiopathologic processes, such as, inflammation, oxidative stress, apoptosis and vascular dysfunction, are involved in the pathogenesis of CI (Dojo Soeandy et al. 2020; Surinkaew et al. 2020; Morris-Blanco et al. 2021). Recently, angiogenesis, which is regulated by a large number of factors, such as vascular endothelial growth factor (VEGF), and fibroblast growth factor 2 (FGF2), has become a hot spot in cerebrovascular disease studies, and enhancing angiogenesis in ischaemia brain tissue might be an effective method for improving blood supply in the brain (Chan et al. 2020; Li et al. 2020). However, the pathogenesis of CI is complicated, and an effective intervention method to prevent or cure the disease has not yet found (Reis et al. 2017). It is a critical to explore an effective multi-target drug to prevent or ameliorate cerebral I/R injury.

Myrtenol is a bicyclic alcohol monoterpene which was found in essential oils of several medicinal plants, such as *Myrtus communis* L. (Myrtaceae), *Rhodiola rosea* L. (Crassulaceae) (Rosenroot), etc. (Rajizadeh et al. 2019). Several reports have confirmed that myrtenol has anxiolytic, antinociceptive, anti-inflammatory, anticancer, antioxidant, and neuroprotectant properties (Rajizadeh et al. 2019; García et al. 2020; Heimfarth et al. 2020). Myrtenol has been used for treatment of anxiety, gastrointestinal pain, inflammations and infections (Moreira et al. 2014; Viana et al. 2016; Gomes et al. 2017). The protective effect of myrtenol against myocardial I/R injury has been demonstrated (Britto et al. 2018). Although multiple biological actions of myrtenol have been reported, there are no studies on whether the myrtenol is an effective multi-target drug to improve cerebral I/R injury.

In the present study, rats with focal cerebral I/R injury were used to investigate the protective effect of myrtenol against cerebral I/R injury and its underlying mechanism.

## Materials and methods

### Animals

Adult male Sprague-Dawley (SD) rats weighing 220–300 g were provided by Laboratory Animal Centre of Shanghai, Chinese Academy of Sciences, and housed in standard cages under controlled temperature of 23 ± 2 °C and a 12-h light/dark cycle, with free access to water and food. All rats were acclimated for 5 days before experimental manipulation. All animal experiments were conducted in accordance with the National Institute of Health Guideline for the Care and Use Committee of Luohe Central Hospital.

### Groups and drug administration

**Figure 1.**
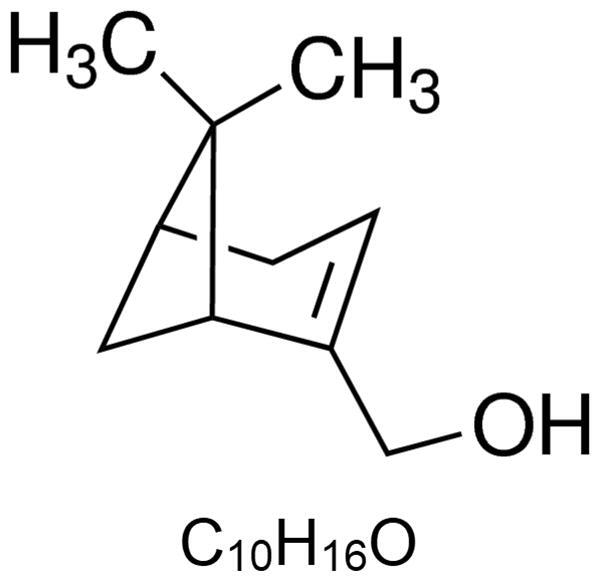
Chemical structure of myrtenol.

Myrtenol was purchased from Sigma-Aldrich Corporation (W343900, CAS-No.19894-97-4, purity ≥95%; Figure 1). Seventy-five SD rats were randomly divided into five groups (*n* = 15): sham group: rats were treated with saline but without middle cerebral artery occlusion (MCAO). MCAO group: rats were given with saline alone with cerebral ischaemia/reperfusion (I/R) surgery. MCAO + Myr groups: rats were intraperitoneally injected with 10, 30, or 50 mg/kg myrtenol after cerebral I/R surgery, respectively (Britto et al. 2018), once daily for seven consecutive days. The remaining 60 SD rats were randomly divided into four groups (*n* = 15): sham group, MCAO group, MCAO + Myr (50 mg/kg) group: rats were treated as above, MCAO + Myr + U0126 group: rats were intracerebroventricularly injected with 10 μL U0126 (Cell Signalling Technology, USA), a highly selective inhibitor of MEK (Ahnstedt et al. 2015), into the cerebral ischaemia side at 30 min prior to myrtenol treatment, once daily for 7 consecutive days. The intracerebral ventricular injection and the dose of U0126 were based on the work of Ye et al. (2018).

### Focal cerebral I/R model

The rat model of focal cerebral I/R by performing MCAO in the left hemisphere, also known as MCAO rats, as previously described (Liu et al. 2018a). All rats were anaesthetized with 10% chloral hydrate by intraperitoneally injection. In brief, the left common carotid artery (CCA) was separated carefully, the external carotid artery (ECA) and internal carotid artery (ICA) were exposed gently. A 3.0 monofilament suture was inserted to cut off the origin of the MCA after clipped the ECA. After 90 min of ischaemia, the monofilament was removed, inducing cerebral reperfusion. Laser Doppler Flowmetry (LDF, PerFlux 5000 Perimed Co., China) was applied to monitor the regional cerebral blood flow (rCBF) during the surgical procedure and at the reperfusion as described previously (Wild et al. 2000). A successful MCAO model was accepted when rCBF < 20% and recovered to higher than 80% of baseline. A total of 135 rats were included in the present experiments. The rats in sham group underwent the same surgery, except that the filament was inserted to cut off the origin of the MCA. The cardiovascular rate and rectal temperature were monitored and maintained during the surgical procedure.

### Neurological deficits evaluation

Twenty-four h after administration, the neurological deficits score of all rats were estimated by a researcher blinded, according to the previous method described by Garcia et al. (1995). The scores were evaluated from motor function and sensory function with minimum neurological deficits score 3 and the maximum 18.

### Diving platform experiment and Y-maze test

The memory and learning capacity of rats in each group were evaluated by diving platform experiment and Y maze test. The diving platform instrument (10 cm × 10 cm × 60 cm) consisted of a reflex box divided into 5 rooms by black plastic board. Copper shutter at 0.5 cm intervals connected with a 36 V electrical current were placed on the bottom of the diving platform instrument. An insulated platform (4.0 cm in diameter, 4.0 cm in height) was placed in the back left corner of each room. Y-maze was consisted of three arms (regions I-III, 30 cm l × 8 cm w × 20 cm h), with the arm at a 120° angle from each other. Three were randomly marked as novel arm, star arm, and other arm. The diving platform experiment and the Y-maze testing were performed as the previously reported (Wen et al. 2014; Liu et al. 2019). The memory differences between the rats of different groups were studied by comparing the frequency of mistake of rats jumping from the insulated platform down to the shutter within 5 min. The times of entries into novel arm for each rat were analysed.

### Measurement of the brain water content

After evaluation of the neurological deficits, all rats were anaesthetized and decapitated rapidly. The brain tissues were removed quickly for the following experiment. The wet weight (A) and dry weight (B, tissues were dried in an oven for 24 h) of brain tissue from three rats in each group were weighed. The brain water content was calculated in accordance with the formula: Brain water content = (A - B)/A × 100%.

### 2,3,5-Triphenyltetrazolium chloride (TTC) staining

TTC staining method was used to measure the cerebral infarct volume. After being rapid-frozen in −20 °C for 20 min, the brain tissues were sliced into 2 mm thick section. And the slices were stained in 2% TTC for 20 min and fixed in 4% paraformaldehyde buffer. The infarcted brain tissue appeared white, whereas the normal tissues showed a red colour. The sections were photographed and the infarct volumes were measured using Image J software.

### Western blot

Western blotting was used to detect the expression of proteins involved in apoptosis, angiogenesis and the MEK/ERK signalling pathway at 24 h after administration. Total protein was extracted using protein extraction kit, according to the manufacturer’s instructions and the protein concentration were measured with BCA method. Protein samples (40 μg) were subjected to 10% SDS-polyacrylamide gels electrophoresis (SDS-PAGE) to separate. Then transferred to poly-vinylidnene fluoride (PVDF, Millipore) membranes and blocked in PBST solution with 5% non-fat milk. The membranes were incubated in primary antibodies against brain derived neurotrophic factor (BDNF), nerve growth factor (NGF), Bax, Bcl-2, VEGF, FGF2, MEK1/2, p-MEK1/2, ERK1/2, p-ERK1/2 (1:2000 dilution, Proteintech, USA) at 4 °C overnight. Secondary antibodies: HRP-conjugated anti-mouse or anti-rabbit IgG (1:5000, Proteintech, USA) were incubated 1 h at 37 °C. Finally, the enhanced chemiluminescent reagent (Thermo Fisher, USA) was added in the membranes and band intensity signals were observed. GAPDH was used as an internal reference, and the optical densities of protein bands were analysed by Image J software to represent the relative expression of target protein.

### Haematoxylin eosin (HE) staining

After completing neurological deficits evaluation, the rats were decapitated under deep anaesthesia. The brains were rapidly removed, the hippocampus was separated, fixed in 10% formalin solution and embedded in paraffin. Coronal sections (4 μm) were obtained and stained with haematoxylin-eosin (HE) solution for the histopathological examination. The scores of histopathological damages were determined as follows: 0, no morphological damage; 1, (slight) edoema or dark neurons; 2, (moderate), edoema or haemorrhages; 3, (severe) local necrosis.

### Terminal deoxynucleotidyl transferase dUTP nick end-labelling (TUNEL) assay

Apoptosis was examined by TUNEL assay. The histological sections (4 μm) were obtained from the paraffin-embedded brains. TUNEL assay was performed using an *in situ* apoptosis detection kit, in accordance with the manufacturer’s instruction. The apoptosis cells were identified as cells with brown-stained nuclei. The number of TUNEL-positive cells was counted in each single visual field and the percentage was calculated as follows: TUNEL-positive cells (%) = (the numbers of TUNEL-positive cells/all cells in a single visual field) × 100%.

### Immunohistochemical staining (IHC)

The expression of vascular endothelial growth factor (VEGF) in cortical penumbra was detected by IHC staining. Paraffin-embedded brain sections were dewaxed, rehydrated, treated with 0.3% H_2_O_2_ for 10 min, and blocked with 5% goat serum for 1 h at 37 °C. Then, the sections were incubated with an anti-VEGF mouse monclonal antibody (1:200, Abcam, USA) at 4 °C overnight. After washing, the secondary antibody: Goat Anti-Mouse HRP-IgG (1:1000) were incubated for 2 h at room temperature. After washing, DAB solution was added into sections to provide the staining for 5 min. And sections were counterstained with haematoxylin and observed under a light microscope. The VEGF-positive cells were characterised by brown granules. The expression of VEGF was represented by the percentage of positive cells in each view at 200× magnification. Five fields were randomly selected in each section.

### Caspase-3 activity assay

Caspase-3 activity of tissue homogenate was measured by Caspase 3 Assay Kit, colorimetric (Sigma-Aldrich, Germany), according to the manufacturer’s protocol.

### Statistical analysis

The data were analysed by SPSS19.0 software. Measurement data were presented as means ± standard deviation from at least three repeated experiments, and the comparison among multiple groups were analysed by one-way analysis of variance, between-group differences were detected by Tukey’s *post hoc* test. *p* < 0.05 was considered the significant difference.

## Results

### Myrtenol improved neurological function and cerebral infarction of MCAO rats

**Figure 2.**
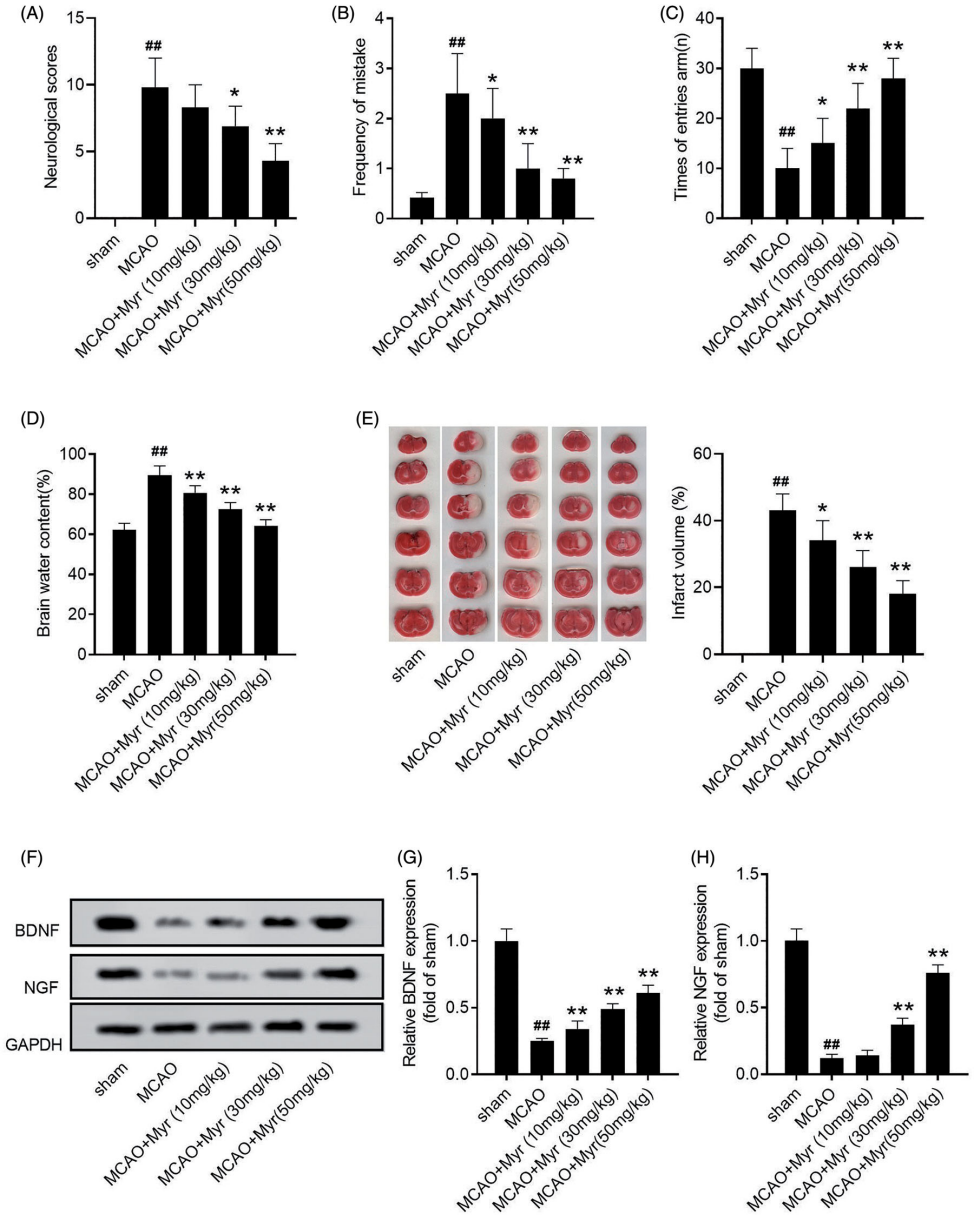
Myrtenol improved neurological function and cerebral infarction of MCAO rats. (A) The neurological score of rats in each group at 24 h after administration (*n* = 15). (B) The frequency of mistakes by rats in each group was detected by diving platform experiment (*n* = 15). (C) The times of entries arm of rats in each group was detected by Y-maze testing (*n* = 15). (D) The brain water content of rats in each group (*n* = 5). (E) Effect of myrtenol on the cerebral infarct volume of rats in each group was measured by TTC staining (*n* = 4). (F-H) The expression of BDNF and NGF in brain tissues of rats in each group was detected by western blot (*n* = 6). Data were presented as the mean ± SD of at least three repeated experiments. ^##^*p* < 0.01, compared with the sham group; *p < 0.05, **p < 0.01, compared with the MCAO group. Except for the sham group, all rats in other groups were constructed for cerebral I/R injury by MCAO. Sham group and MCAO group, given saline after cerebral I/R surgery. MCAO + Myr groups, administered 10, 30, or 50 mg/kg myrtenol after cerebral I/R surgery, respectively. All rats were treated once daily for seven consecutive days.

Rats subjected to 90 min of MCAO followed by reperfusion were used to simulate the pathology of cerebral I/R injury. To assess the effects of myrtenol on cerebral I/R injury, myrtenol (10, 30, or 50 mg/kg) were intraperitoneally injected into MCAO rats. MCAO rats showed prominent neurological deficit, while myrtenol could ameliorate the neurological function of MCAO rats (*p* < 0.05 vs. sham group; Figure 2(A)). The memory and learning capacity were also assessed using diving platform experiment and Y-maze test. As shown in Figure 2(B,C), myrtenol could decrease the frequency of mistakes in the diving platform experiment (2.02 ± 0.98% for 10 mg/kg myrtenol group, 1.07 ± 0.93% for 30 mg/kg myrtenol group, 0.82 ± 0.21% for 50 mg/kg myrtenol group; *p* < 0.05), whereas increase the times of entries arm in Y-maze test of MCAO rats (15.03 ± 5.04% for 10 mg/kg myrtenol group, 22.14 ± 4.96% for 30 mg/kg myrtenol group, 27.97 ± 4.02% for 50 mg/kg myrtenol group; *p* < 0.05). Additionally, myrtenol could reduce cerebral edoema in MCAO rats, which was confirmed by the reduced brain water content (*p* < 0.05 vs. sham group; Figure 2(D)). Moreover, TTC staining was performed to assess the cerebral infarction of MCAO rats. As expected, myrtenol treatment markedly attenuate the cerebral infarct volume of MCAO rats (34.11 ± 6.02% for 10 mg/kg myrtenol group, 26.06 ± 5.33% for 30 mg/kg myrtenol group, 18.16 ± 4.08% for 50 mg/kg myrtenol group; *p* < 0.05; Figure 2(E)).

We also evaluated the expression of brain-derived neurotrophic factor (BDNF) and nerve growth factor (NGF) in hippocampus, which are considered to be the most important neurotrophins closely related to cognitive function. Western blot assay showed that MCAO could down-regulated the expression of BDNF and NGF in brain tissues of rats, while myrtenol treatment could restore the expression of BDNF and NGF (*p* < 0.05 vs. sham group Figure 2(F–H)). The results suggested that myrtenol could improve the neurological function and reduced the cerebral infarct volume in the rats with cerebral I/R.

### Myrtenol improved hippocampus and reduced cell apoptosis in MCAO rats

**Figure 3.**
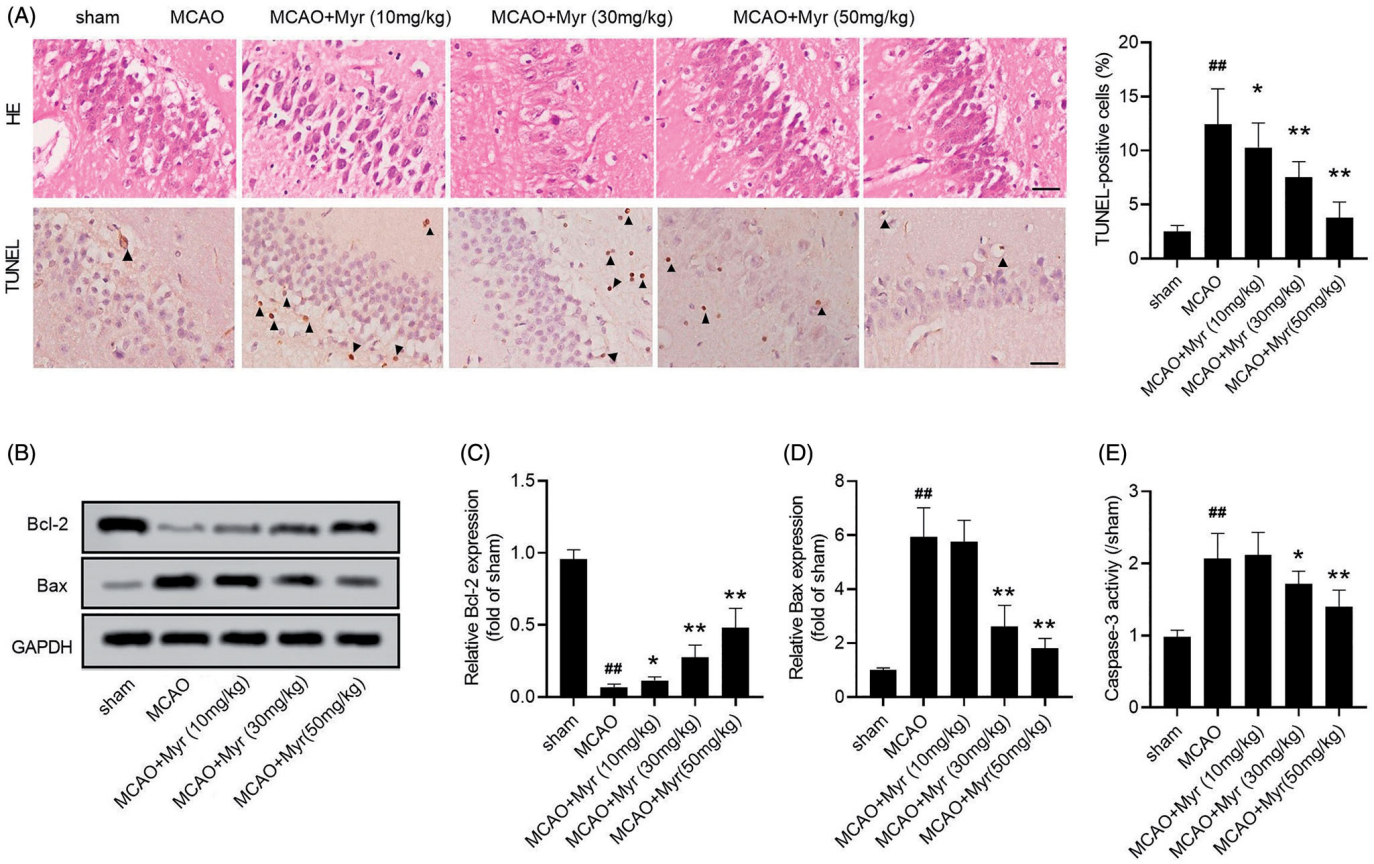
Myrtenol improved hippocampus damage and reduced cell apoptosis in MCAO rats. (A) Histological damage and apoptosis of hippocampus were analysed using HE staining and TUNEL assay (*n* = 6). (B–D) The expression of apoptosis marker protein, Bcl-2 and Bax, in hippocampus of rats in each group was detected by western blot (*n* = 6). (E) The caspase-3 activity in hippocampus of rats in each group (*n* = 6). Data were presented as the mean ± SD of at least three repeated experiments. ^##^*p* < 0.01, compared with the sham group; *p < 0.05, **p < 0.01, compared with the MCAO group.

Cerebral ischaemia is often accompanied by morphological and functional changes in the hippocampus (Li et al. 2020). Therefore, the histopathological damage of hippocampus was evaluated by HE staining. In the sham group, the cell outline was clear and structure was compact, the nucleolus was clearly visible, without interstitial. However, cells were arranged sparsely and structure was disorder in the MCAO group. Additionally, nerve cells swelling, interstitial oedema, nerve cell deformation, nuclear pyknosis and tissue necrosis were also found in the hippocampus of MCAO rats. Myrtenol treatment could improve nerve cell swelling, interstitial edoema, cell degeneration and necrosis (Figure 3(A)). TUNEL staining assay revealed that cell apoptosis in the hippocampus of MCAO rats was obviously increased (Figure 3(A)). Simultaneously, western blot assays also showed that the expression of Bcl-2 was down-regulated, while Bax was up-regulated in the hippocampus of MCAO rats (*p* < 0.05; Figure 3(B–D)). Additionally, the caspase-3 activity was also increased in MCAO rats (2.07 ± 0.21%-fold change for 10 mg/kg myrtenol group, 1.78 ± 0.18%-fold change for 30 mg/kg myrtenol group, 1.36 ± 0.19%-fold change for 50 mg/kg myrtenol group; *p* < 0.05; Figure 3(E)). However, myrtenol treatment could reduce cell apoptosis in brain tissues induced by MCAO (Figure 3(A–E)). The data indicated that myrtenol suppressed the histopathological damage and apoptosis of hippocampus induced by MCAO *in vivo*.

### Myrtenol promoted angiogenesis in MCAO rats

**Figure 4.**
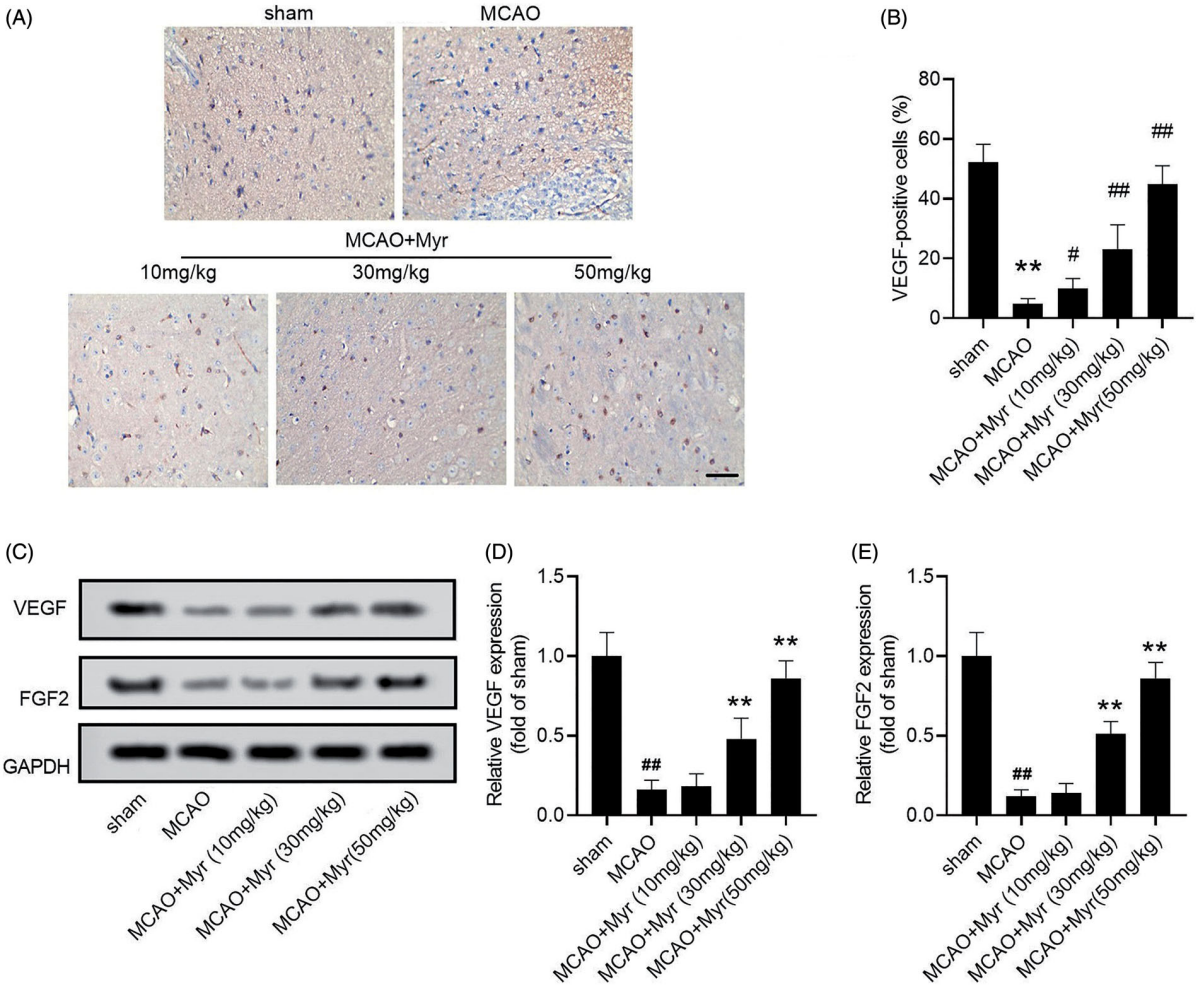
Mytenol promoted angiogenesis in MCAO rats. (A–B) VEGF expression in ischaemic penumbra were determined by IHC staining (*n* = 6). (C–E) The relative expression of VEGF and FGF2 protein was detected by western blot (*n* = 6). Data were presented as the mean ± SD of at least three repeated experiments. ^##^*p* < 0.01, compared with the sham group; *p < 0.05, **p < 0.01, compared with the MCAO group.

Angiogenesis in ischaemic penumbra is one of the important early events after cerebral ischaemia (Taguchi et al. 2004). To further evaluate whether myrtenol affects angiogenesis in ischaemic penumbra of MCAO rats, we detected the expression of pro-angiogenic factors VEGF and FGF2. The IHC showed that the positive expression of VEGF in ischaemic penumbra of MCAO rats was lower than that in the sham group, while myrtenol treatment could increase the positive staining of VEGF to a certain extent (Figure 4(A,B)). Furthermore, western blot assay also showed increased expression of VEGF and FGF2 in MCAO rats (*p* < 0.05 vs. sham group), whereas myrtenol treatment could markedly increase the expression of VEGF and FGF2 in brain tissues of MCAO rats (*p* < 0.05 vs. MCAO group; Figure 4(C–E)). The results revealed that myrtenol could up-regulate the expression of VEGF and FGF2 to promote angiogenesis.

### Myrtenol activated ERK1/2 signalling pathway in MCAO rats

**Figure 5.**
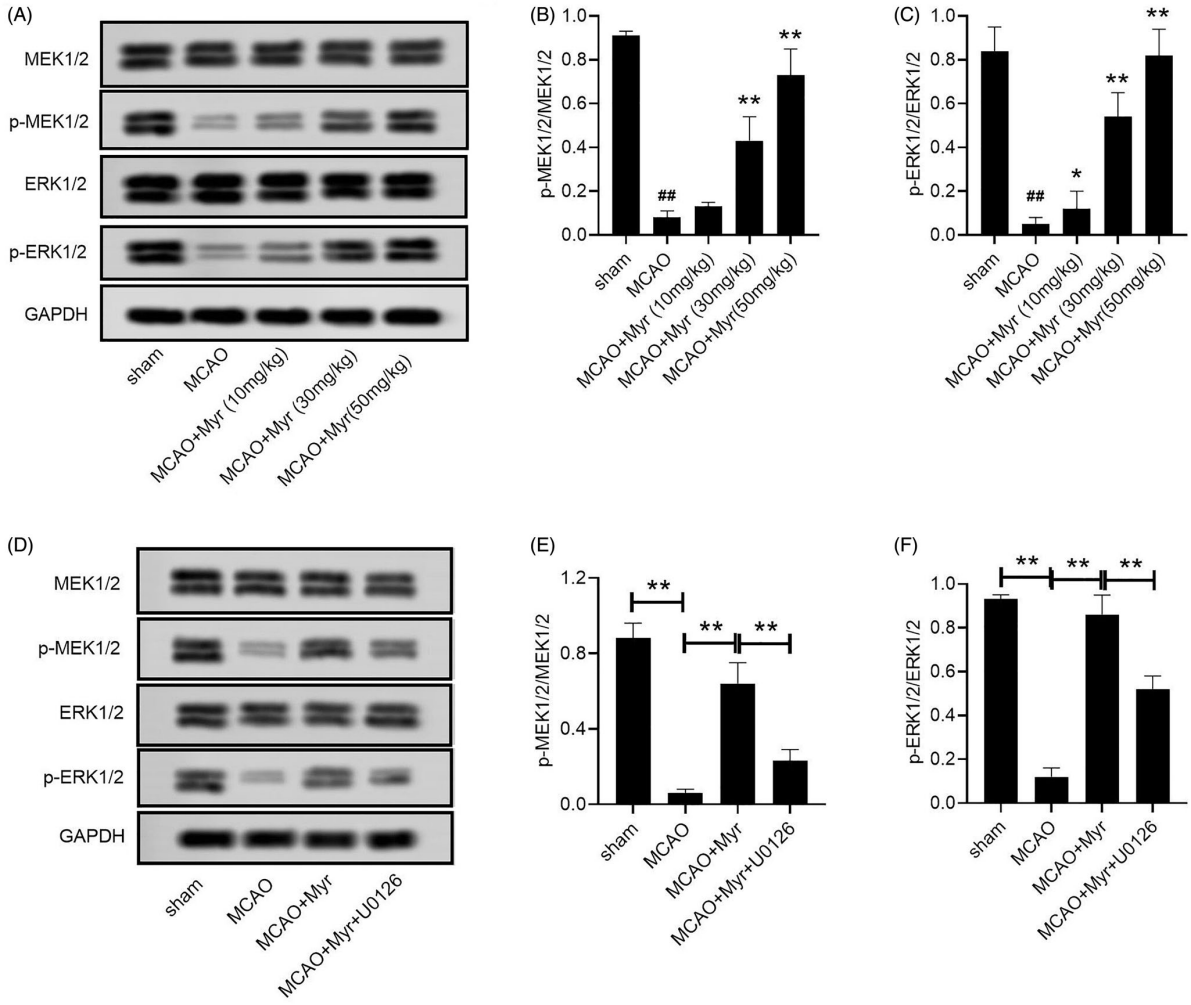
Mytenol activated ERK1/2 signalling pathway in MCAO rats. (A) MEK1/2, p-MEK1/2, ERK1/2, p-ERK1/2 expression in brain tissues were detected by western blot. (B–C) The relative expression ratio of p-MEK1/2/MEK1/2, p-ERK1/2/ERK1/2. (D) MEK1/2, p-MEK1/2, ERK1/2, p-ERK1/2 expression in brain tissues were detected by western blot. (E-F): MEK1/2, p-MEK1/2, ERK1/2, p-ERK1/2 expression in brain tissues were detected by western blot. *n* = 6. Data were presented as the mean ± SD of at least three repeated experiments. ^##^*p* < 0.01, compared with the sham group; *p < 0.05, **p < 0.01, compared with the MCAO group.

The ERK1/2 signalling pathway has been identified as a potentially important role in cerebral ischaemia reperfusion injury (Shi et al. 2021). Western blot assay confirmed the inhibition of the ERK1/2 pathway in the brain tissues of MCAO rats, which was manifested by increased phosphorylation of MEK1/2 and ERK1/2 (*p* < 0.05 vs. sham group; Figure 5(A–C)). Myrtenol treatment could increase the phosphorylation of MEK1/2 and ERK1/2 (*p* < 0.05 vs. MCAO group; Figure 5(A–C)), suggesting that myrtenol activated the ERK1/2 signalling pathway. We also performed rescue experiments by using U0126, the specific inhibitor of ERK1/2 pathway. The activation of myrtenol on the ERK1/2 pathway could be eliminated by U0126 (*p* < 0.05 vs. MCAO + Myr group; Figure 5(D–F)).

### U0126 reversed the effect of myrtenol on the improvement of brain damage and angiogenesis in rats with cerebral I/R

**Figure 6.**
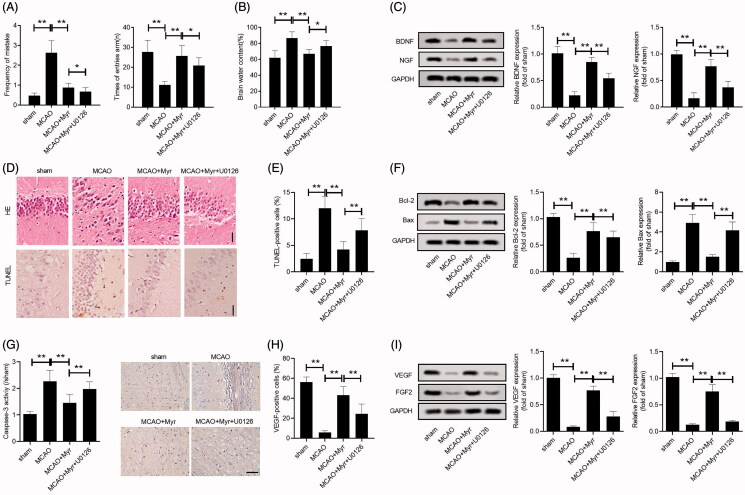
U0126 reversed the effect of myrtenol on the improvement of brain damage and angiogenesis in MCAO rats. (A) Frequency of mistakes, times of entries arm of rat in each group were detected by diving platform experiment, Y-maze testing (*n* = 15). (B) Brain oedema was assessed by brain water content (*n* = 5). (C) Relative expression of BDNG and NGF in brain tissues was detected by western blot (*n* = 6). (D–E) Histological damage and apoptosis of hippocampus were analysed using HE staining and TUNEL assay (*n* = 6). (F) Relative expression of Bcl-2, Bax was detected by western blot (*n* = 6). (G) The caspase-3 activity. (H) IHC staining showed the VEGF positive cells (*n* = 6). (I) Relative expression of VEGF and FGF2 was detected by western blot (*n* = 6). Data were presented as the mean ± SD of at least three repeated experiments. **p < 0.01. Except for sham group, all rats in other groups were constructed for cerebral I/R injury by MCAO. sham group and MCAO group, given with saline after cerebral I/R surgery. MCAO + Myr group, administered 50 mg/kg myrtenol after cerebral I/R surgery; MCAO + Myr + U0126 group, injected with U0126 at 30 min prior to myrtenol treatment. All rats were administered once daily for 7 consecutive days.

Next, we explored whether myrtenol improved brain damage and angiogenesis by activating the ERK1/2 signalling pathway. Diving platform experiment and Y-maze test showed that U0126 could suppress the improvement of myrtenol on memory and learning capacity of MCAO rats (*p* < 0.05 vs. MCAO + Myr group; Figure 6(A)). Similarly, U0126 also restrained the inhibitory effect of myrtenol on cerebral edoema (*p* < 0.05 vs. MCAO + Myr group; Figure 6(B)). Additionally, the increased expression of BDNF and NGF in MCAO rats treated with myrtenol could be abolished by U0126 pre-treatment (*p* < 0.05 vs. MCAO + Myr group; Figure 6(C)). The HE staining also showed that the U0126 could eliminate the improvement effect of myrtenol on brain tissue damage in MCAO rats (Figure 6(D)). Similarly, TUNEL assay also showed that U0126 treatment could reverse the inhibitory effect of myrtenol on MCAO-induced apoptosis (Figure 6(D and E)). Consistently, the inhibition of Bax expression and caspase-3, as well as the promotion of Bcl-2 expression induced by myrtenol treated in MCAO rats could be eliminated by U0126 (*p* < 0.05 vs. MCAO + Myr group; Figure 6(F and G)). Additionally, U0126 also eliminated the promotion of myrtenol on angiogenesis in the brain tissues of MCAO rats, which was demonstrated by inhibiting the expression of VEGF and FGF2 (*p* < 0.05 vs. MCAO + Myr group; Figure 6(H and I)). Collectively, U0126 reversed the protective effect of myrtenol on cerebral I/R injury, which was associated with the improvement of brain damage and angiogenesis.

## Discussion

Cerebral I/R injury often accompany by high mortality and long-term disabilities. As a member of bicyclic monoterpene alcohols family, the traditional medicine myrtenol presented important biological properties and therapeutic potential, such as anti-inflammatory, antioxidant (Viana et al. 2019). However, the role of myrtenol in cerebral I/R injury is unclear. It is worth noting that Britto et al. (2018) revealed myrtenol protected heart against myocardial I/R injury by anti-apoptotic and antioxidant. In this study, the results showed that myrtenol treatment could improve neurological function, cerebral infarction and brain tissue pathological damage of MCAO rats. Additionally, the protective effect of myrtenol on MCAO rats was related to its inhibition of hippocampal neuronal apoptosis and promotion of cerebral angiogenesis, which may be achieved by activating ERK1/2 signalling pathway. Our findings provided evidence indicating that myrtenol may become a promising drug for the therapy of CI injury.

Apoptosis is a vital pathophysiological mechanism associated with I/R, and reperfusion could accelerate the apoptotic death process induced by ischaemia (Villa et al. 2003). Compelling findings indicate the inhibition of apoptosis as a key protective mechanism against cerebral I/R injury (Baldrati et al. 2020; Yu et al. 2020). Wen et al. (2019) demonstrated that N-Myc downstream-regulated gene 4 (NDRG4) protected cerebral IR injury by inhibiting cell apoptosis and regulated cerebral cell apoptosis by increasing BDNF expression. In this study, the results also revealed that the apoptosis of hippocampal neurons was significantly increased after cerebral I/R injury, and myrtenol could suppress the apoptosis in brain tissues. Bcl-2 family related proteins, including anti-apoptotic proteins (such as Bcl-2, Bcl-xL) and pro-apoptotic proteins (such as Bax, Bcl-xS), has been proven to play an important role in the execution of apoptosis (Sergio et al. 2018). It has been confirmed that Bcl-2 can inhibit oxide-induced apoptosis, while Bax promotes the release of cytochrome C and activates caspase-3, which is considered to be the ultimate executor of apoptosis (Abu Zeid et al. 2018). In addition, the increase in Bcl-2 expression and the decrease in Bax expression in the hippocampus after ischaemia can protect against cerebral ischaemic injury by reducing neuronal cell apoptosis (Yi et al. 2020). We also found that myrtenol prevented the down-regulation of Bcl-2 and the upregulation of Bax, as well as the activity of caspase-3 induced by MCAO. Therefore, the inhibitory effect of myrtenol on neuronal apoptosis in MCAO rats may involve its regulation of apoptosis-related proteins.

Angiogenesis is a process of growing new capillaries from pre-existing vessels during some pathophysiological conditions, such as tumour growth, tissue ischaemia (Liu et al. 2014). Emerging evidences have shown that angiogenesis is the most effective way to restore blood supply and improve functional recovery of ischaemic brain tissue, ameliorating cerebral I/R injury (Lapi and Colantuoni 2015; Peng et al. 2019). As a pro-angiogenic factor, VEGF is produced and secreted by many neurovascular cells in brain and considered as a central mediator in post-ischaemic angiogenesis (Liu et al. 2018b). VEGF increases the expression of other pro-angiogenic factors, including fibroblast growth factor 2 (FGF2), which binds to FGF receptors playing a crucial role in the angiogenic process (Liu and Chen 1994; Seo et al. 2013). In this study, we also found that myrtenol promotes angiogenesis, which was demonstrated by the up-regulation of VEGF and FGF2 expression. Relevant studies on drug components to reduce ischaemic stroke by promoting angiogenesis have been widely confirmed. For example, bilobalide benefits post-ischaemia stroke symptoms by promoting angiogenesis and reducing both apoptosis and autophagy (Zheng et al. 2018b), Buyang Huanwu Decoction exerted neuroprotection targeting angiogenesis through the up-regulation of SIRT1/VEGF pathway against cerebral ischaemic injury in rats (Zheng et al. 2018a). We speculated that the improvement effect of myrtenol on cerebral I/R injury may involve its promotion of angiogenesis.

MEK1/2/ERK1/2 signalling pathway participate in cell growth, apoptosis, and involved in the neuroprotection against ischaemia brain damage, likely playing a critical role in recovery of ischaemia injury (Krylatov et al. 2021). Therefore, we investigated whether the ERK1/2 pathway was related to the protective effect of myrtenol in cerebral I/R injury. We found that myrtenol treatment eliminated the inhibitory effect of the ERK1/2 pathway caused by MCAO, which suggested that the protective effect of myrtenol on cerebral I/R injury may be related to the activation of the ERK1/2 pathway. Additionally, the activation effect of myrtenol on the ERK1/2 pathway could be eliminated by U0126. Consistently, U0126 treatment could reverse the neuroprotective effect of myrtenol on MCAO rats to a certain extent. The ERK1/2 pathway regulates cell apoptosis by regulating Bcl-2 family proteins (Ren et al. 2020). The phosphorylation of ERK1/2 can cause the phosphorylation of Bad protein and make it lose its ability to bind to Bcl-2, which leads to the combination of Bcl-2 and Bax to form a dimer and ultimately increase the cell's resistance to apoptosis. Besides, the induction of ERK1/2 activation is related to the activation of the caspase-8/caspase-3 pathway (Snyder et al. 2010). Moreover, The ERK1/2 pathway is necessary for angiogenesis stimulated by multiple growth factors (such as VEGF and FGF) (Dai et al. 2009). Song et al. (2012) found that inhibition of VEGF receptor-mediated ERK1/2 signalling pathway can inhibit breast cancer cell proliferation and angiogenesis *in vitro*. Based on these studies, we speculated that the anti-apoptotic and pro-angiogenesis effects of myrtenol in cerebral I/R injury were achieved to some extent by activating the ERK1/2 pathway.

## Conclusions

The data demonstrated that the ERK1/2 signalling pathway contributed to the protective effects of myrtenol against cerebral I/R injury in rats, which was associated with the attenuation of brain damage and angiogenesis. These findings provided further insight into the specific mechanisms of how myrtenol exerted its protective effects on cerebral I/R injury and also provided more theoretical basis for the clinical application of myrtenol.
